# The Imprint of Extreme Climate Events in Century-Long Time Series of Wood Anatomical Traits in High-Elevation Conifers

**DOI:** 10.3389/fpls.2016.00683

**Published:** 2016-05-18

**Authors:** Marco Carrer, Michele Brunetti, Daniele Castagneri

**Affiliations:** ^1^TeSAF Department, Università degli Studi di PadovaPadova, Italy; ^2^Institute of Atmospheric Sciences and Climate, National Research CouncilBologna, Italy

**Keywords:** cell diameter, cell number, cell-wall thickness, dendroanatomy, extreme climate events, tree ring, xylem anatomy

## Abstract

Extreme climate events are of key importance for forest ecosystems. However, both the inherent infrequency, stochasticity and multiplicity of extreme climate events, and the array of biological responses, challenges investigations. To cope with the long life cycle of trees and the paucity of the extreme events themselves, our inferences should be based on long-term observations. In this context, tree rings and the related xylem anatomical traits represent promising sources of information, due to the wide time perspective and quality of the information they can provide. Here we test, on two high-elevation conifers (*Larix decidua* and *Picea abies* sampled at 2100 m a.s.l. in the Eastern Alps), the associations among temperature extremes during the growing season and xylem anatomical traits, specifically the number of cells per ring (CN), cell wall thickness (CWT), and cell diameter (CD). To better track the effect of extreme events over the growing season, tree rings were partitioned in 10 sectors. Climate variability has been reconstructed, for 1800–2011 at monthly resolution and for 1926–2011 at daily resolution, by exploiting the excellent availability of very long and high quality instrumental records available for the surrounding area, and taking into account the relationship between meteorological variables and site topographical settings. Summer temperature influenced anatomical traits of both species, and tree-ring anatomical profiles resulted as being associated to temperature extremes. Most of the extreme values in anatomical traits occurred with warm (positive extremes) or cold (negative) conditions. However, 0–34% of occurrences did not match a temperature extreme event. Specifically, CWT and CN extremes were more clearly associated to climate than CD, which presented a bias to track cold extremes. Dendroanatomical analysis, coupled to high-quality daily-resolved climate records, seems a promising approach to study the effects of extreme events on trees, but further investigations are needed to improve our comprehension of the critical role of such elusive events in forest ecosystems.

## Introduction

It has become manifest that climate change is producing significant effects on natural systems and human society worldwide. This condition will likely worsen as, despite different emission scenarios, all current projections indicate a warming trend in the future, associated to a corresponding change in the frequency, severity, and nature of extreme events (IPCC, 2013). Small changes in the mean or variance of a climate variable may lead to disproportionally large changes in the frequency of extremes, representing a severe challenge for living organisms to respond adaptively (Gutschick and BassiriRad, 2003). These events are now recognized as major drivers of current and future ecosystem dynamics (Smith, 2011; Frank et al., 2015). Biological responses to extreme weather events can vary, and even be reversed among different species or growth stages in the same habitat, but the impact can be pervasive and propagates through the ecosystem with a cascade of side effects (Parmesan et al., 2000).

Characteristic infrequency and stochasticity challenges investigations of extreme events, and is the reason for the lack of a comprehensive, precise and biologically meaningful definition of them (Gutschick and BassiriRad, 2003). Besides, extreme events occur in a wealth of divergent types (e.g., heat waves, hurricanes, droughts, ice storms, etc.), at multiple time scales, are highly context dependent, and their effects, rather than linear and monotonic, are usually non-linear and threshold based (Knapp et al., 2008; Smith, 2011; Bahn et al., 2014; Frank et al., 2015). This calls for research focused on extreme events and their consequences in different ecosystems at multiple time, spatial and magnitude scales by collecting evidence in both natural and controlled conditions (Jentsch et al., 2007; Reyer et al., 2015). Up to now, most studies have been conducted in controlled environments, on short-lived species or early life stages (Marchand et al., 2005; Wang et al., 2008; De Simon et al., 2013), or tracking the effects of a single event (Ciais et al., 2005; Piayda et al., 2014; Balducci et al., 2015; Julio Camarero et al., 2015). Due to differences in longevity among growth forms and climatic sensitivities among early and adult life stages, and the idiosyncratic behavior of any extreme events, it is difficult to extrapolate these findings to forest ecosystems (Teskey et al., 2015). To assess the effect of extreme climate events on trees and forests our inferences should be based on long-term observations just to cope with the typical life cycle of these organisms and the paucity of the events themselves (Jentsch et al., 2007; Anderegg et al., 2015).

To date, working with time series seems the most straightforward approach to deal with the effect of extreme climate events on long-lived organisms such as trees (Jentsch et al., 2007). Within this context, tree-ring analysis can deliver most of its potential considering the intrinsic association with annually resolved and absolutely dated chronologies of different tree-ring parameters. Building up to millennia-long tree-ring series allows a vast time dimension, an essential perspective to soundly assess the effects and potential changes in frequency, intensity, and nature of extreme events (Babst et al., 2012). Actually, the study of extreme event effects on tree-ring width and structure has a long tradition in tree-ring science, thanks to relevant tree-ring features that mark a direct signature of climate extremes such as frost rings (Rhoads, 1923; Harris, 1934; Lamarche and Hirschboeck, 1984), intra annual density fluctuations (Schulman, 1938), white- or blue-rings (Waito et al., 2013; Piermattei et al., 2015), resin-duct density (Wimmer and Grabner, 1997) – see Schweingruber et al. (1990) and Schweingruber (1996) for a thorough review. However, despite the wealth of studies there are still two major facets that could be improved: (i) the lack of a rigorous causal relationship of climate extremes on wood structure, which has led to an anecdotal rather than systematic analysis in most of the investigations and (ii) the time resolution, still mostly bounded at yearly or monthly level at best, leaving the extreme events occurring at shorter time scales largely unscrutinized.

Dendroanatomy, the study of wood-anatomical traits with a dendrochronological approach, has been expanding recently thanks to technological and technical advances (Wegner et al., 2013; von Arx and Carrer, 2014). Working at anatomical level can provide new clues for understanding climate extreme effects on tree growth and functioning: first, detailed assessment of xylem traits gets closer to the functional and physiological tree aspects, making the link between pattern and process more apparent. Second, investigating intra-ring anatomical traits, the time resolution of the analyses can potentially significantly increase compared to a typical tree-ring approach, i.e., reaching the sub-monthly level. In this study, we test the potential of dendroanatomy to deal with extreme climate events. In particular, our research question was: is it possible to detect the imprint of past temperature extremes occurred during the growing season at wood anatomical level? To address this question, we collected wood samples from two high-elevation conifers (*Larix decidua* and *Picea abies*) in the Alps and analyzed some xylem anatomical parameters, namely the number of tracheids per ring, their diameter and wall thickness, for any potential associations with extreme temperature events.

## Materials and Methods

### Study Site

Analyses were performed on two conifer species: *Picea abies* (L.) Karst. (Norway spruce), evergreen, and *Larix decidua* Mill. (European larch), deciduous. Both the species are widespread in the Alps, and reach the treeline, which in the Eastern Italian Alps occurs at around 2200 m a.s.l. The study site was located at an elevation of 2100 m a.s.l., close to Cortina d’Ampezzo (46°30′ N, 12°07′ E). At the valley bottom, mean annual precipitation is 1080 mm, with a maximum in June. Daily maximum temperature averages 20.8°C during July, and 3.1°C in January (Cortina d’Ampezzo meteorological station, 1275 m a.s.l., 1926–2011).

### Instrumental Climatological Data

The availability of long and reliable temporal series of meteorological variables at a fine space-time resolution is crucial when the analysis target goes beyond the common climate-ring width associations and aims at investigating climate influence on xylem cell structure. However, global or regional climatological datasets frequently lack representativeness at local scale, especially in areas with rugged terrain. We therefore reconstructed climate variability more accurately taking into account the relationship between meteorological variables and the topographical settings of the region. The climate information comes from the daily minimum and maximum temperature series of the Cortina D’Ampezzo station, covering the 1926–2011 period, and from synthetic records of monthly minimum, mean, and maximum temperatures covering the period 1800–2011 reconstructed for the specific site location.

As well as for any other meteorological measure, physical signals in raw temperature data series are often hidden behind non-climatic noise caused mainly by station relocation and changes in instruments, in the environment around the station or in the observing conventions. The noise represented by non-climatic disturbances in the raw data is often of the same order of magnitude as the target climate signal, or even greater. For this reason, data homogenization (i.e., the procedure to remove non-climatic signals) is crucial to ensure the reliability of the dataset in representing the true climatic signal.

The homogenization approach used in this study was the same as that discussed in Brunetti et al. (2006), but adapted to daily resolution. We checked monthly minimum and maximum temperature series of Cortina d’Ampezzo separately, by means of a multiple application of the Craddock test (Craddock, 1979), using as references the nearest series available from Brunetti et al. (2006) and Simolo et al. (2010). Monthly correcting factors were estimated using at least three reference series among the neighboring most correlated ones and performing a trigonometric smoothing of the correcting factors. Daily adjustments were then calculated by fitting a trigonometric function to monthly factors, resulting in 366 daily correcting factors.

Synthetic records of monthly minimum, mean and maximum temperatures covering the period 1800–2011 were reconstructed to be representative of the specific location of the sampled site by means of the anomaly method (New et al., 2000; Mitchell and Jones, 2005) as described in Brunetti et al. (2012). The spatio-temporal structure of the signal of a meteorological variable over a given area can be described by the superimposition of two fields: the climatological normals over a given reference period (i.e., the climatologies), characterized by remarkable spatial gradients, and the departures from them (i.e., anomalies), generally characterized by higher spatial coherence and linked to climate variability. Climatologies and anomalies were reconstructed in a completely independent way from each other and based on different data sets (high spatial density and limited temporal coverage for the climatologies, and low spatial density but long temporal coverage and accurate homogenization for the anomalies). Climatologies and anomalies were reconstructed estimating the local temperature-elevation relationship (Brunetti et al., 2014) and using weighted averages of high-quality and homogenized neighboring series (Brunetti et al., 2006), respectively. Finally, the two fields were superimposed to obtain a temporal series in absolute values representative of the site location.

### Samples Collection and Processing

Two increment cores were extracted at breast height with a Pressler borer from 15 trees of each species. Ring widths were measured to the nearest 0.01 mm using TsapWin (Rinntech, Heidelberg, Germany) and then crossdated to match each tree ring with its year of formation (Stokes and Smiley, 1968; Holmes, 1983).

**FIGURE 1:**
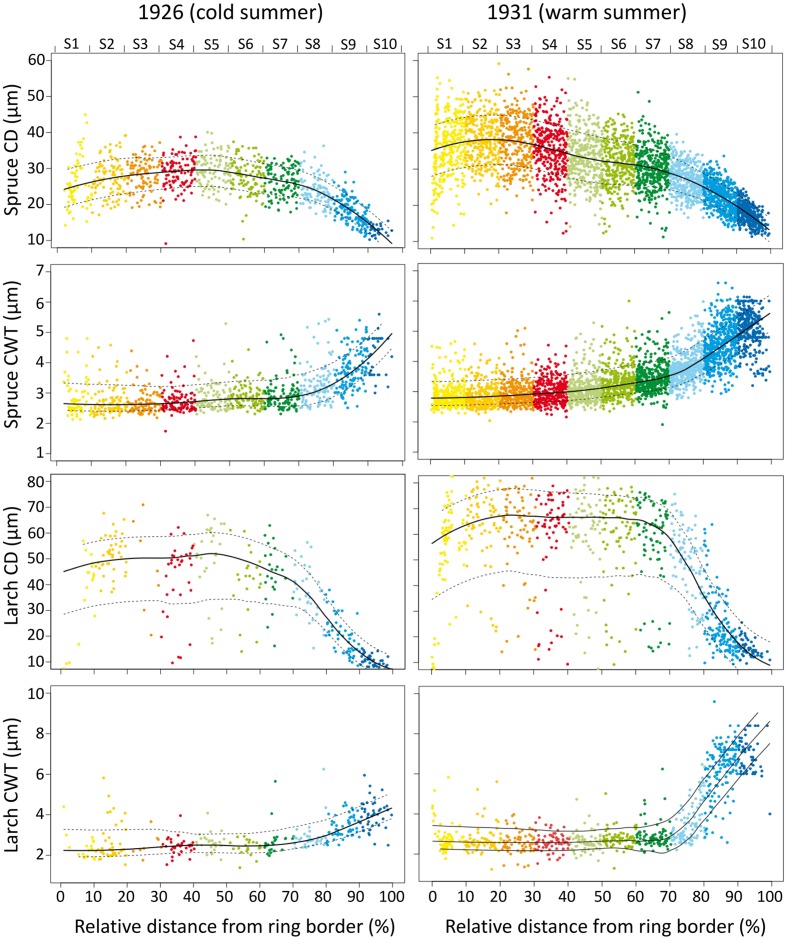
**Tree-ring anatomical profiles during a cold (1926, the first year with an extreme cold June in the 1926–2011 period) and a warm (1931, the first year with an extreme warm June in the 1926–2011 period) year in a spruce (PA04) and a larch (LD04) tree.** *X*-axis represents the relative distance from the ring border, and the corresponding sector (upper axis and different colors). Dots are individual cell values, solid and dotted lines represent the mean and standard deviation values. Note different *Y*-axis scales for the four anatomical traits.

Eight cores for spruce and six for larch (corresponding to 14 trees) were then selected among those without visible faults such as nodes, reaction wood, rotten or missing parts. These cores were split in 4–5 cm long pieces for anatomical measurements. A rotary microtome (Leica, Heidelberg, Germany) was used to obtain 15–20 μm thick transversal micro-sections, which were stained with safranin (1% in distilled water) and fixed on permanent slides with Eukitt (BiOptica, Milan, Italy). Digital images were captured with a light microscope at 40× magnification (Nikon Eclipse 80 mounted with distortion-free lenses), and stitched together with PTGui software (New House Internet Service B.V., Rotterdam, The Netherlands). The images were then processed with the image analysis software ROXAS v2.1 (von Arx and Carrer, 2014). After a brief manual fine-editing to remove objects wrongly identified as cells, the software automatically provided the lumen and wall size together with the relative position within the dated annual ring of each cell in the image. With this information, we divided each ring into 10 sectors of equal width along the tangential direction and created the tree-ring anatomical profiles. These profiles represent the variation of different anatomical parameters within each ring (**Figure 1**). As an example, sector 1 comprises all the cells with a distance from the initial earlywood ring border to 10% of the total ring width. For each sector, we computed (1) mean cell wall thickness (CWT) and (2) mean cell diameter (CD). For each tree, we therefore built 10 CD and 10 CWT time series, which ranged from earlywood to latewood, representing distinct time windows within the growing season. We also computed (3) the total number of cells per ring (CN), and corresponding individual CN series.

**FIGURE 2:**
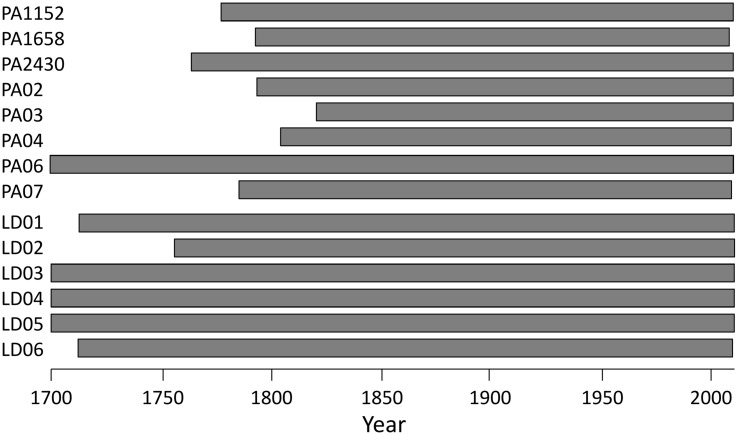
**Time span covered by anatomical series in the sampled trees**.

For each parameter, species and sector, we built mean chronologies by computing the bi-weight robust mean from the detrended individual time series. Indeed, most of the anatomical parameters (**Figure 2**) presented a typical age/size trends (Carrer et al., 2015), which can alter correlations with climate (Cook et al., 1990). Individual series were therefore detrended by fitting a stiff function (power function or 200 years cubic spline with 50% frequency cut-off) to raw data, and dividing observed by expected values.

Some descriptive statistics, within the 1800–2011 common period, have been adopted to compare and describe the resulting chronologies. These are the mean sensitivity (MS) and mean correlation between the series (Rbar) to assess the strength of the high frequency variability within the series and the level of year-by-year growth variations shared by trees of the same species, respectively.

### Anatomical-Traits Response to Climate Extremes

Previous analyses demonstrated that maximum spring-summer temperature is the main climate factor affecting spruce and larch radial growth processes at this elevation (Carrer and Urbinati, 2004; Rossi et al., 2007; Castagneri et al., 2015b). We therefore investigated anatomical-traits response to spring-summer temperature at two distinct scales of detail.

In the first analysis, we investigated how climate extremes, assessed at monthly scale over the period 1800–2011, influence the anatomy of tree rings. We initially explored mean responses to inter-annual climate variability in order to identify the month (or monthly aggregate) when climate has the largest influence on CN, CD, and CWT and computed correlation between chronologies (whole ring CN and the 10 sectors CD and CWT) and temperature from May to October. The monthly period considered largely comprises the beginning and the end of the typical growing season for both species in the area (Rossi et al., 2008). Then, we computed the yearly tree-ring profiles of CD and CWT from the first to the last sector together with the CN value for all the rings. Finally, we contrasted the median CN value and median CD and CWT profiles for all the years in 1800–2011, with those computed for the 10 coldest and 10 warmest years for the month (or monthly aggregate) selected with the correlation analysis. To test for a potential carry over effect, we also considered the anatomical traits in the years following the extreme events.

In the second analysis, using daily temperature records over the period 1926–2011, we were able to refine the analyses to better cope with the short-term climate influence on cell parameters, and to go beyond the artificial aggregation of climate variability into months (Fonti et al., 2013). In particular, we scrutinized how CN, CD, and CWT positive and negative extremes within intra-ring sectors were associated to short-term specific climate conditions, First, we calculated for each parameter the correlation with temperature to identify the period within the season with the highest sensitivity to climate variability. For CD, determined by cell enlargement which lasts 14–25 days in the first earlywood tracheids to very few days in the last cells (Rossi et al., 2008), daily maximum temperature data were averaged over a 15-days moving window shifted at daily step, and running Pearson correlations with CD sector chronologies were computed between May 1st and October 31st of the ring formation year. For CWT, related to the wall thickening phase that lasts up to 40 days in these species (Rossi et al., 2008), the same procedure was applied using 30-days moving windows. Similarly, as CN depends on growth during the whole growing season, 30-days moving windows were used. This allowed identification with 1-day precision, of the 15- or 30-days period when the temperature sensitivity of each anatomical parameter peaks. We then considered the first and last 10 values (extremes in anatomical traits) of CN, CD and CWT, corresponding to the tails of their distributions, and pinpointed the corresponding calendar year. In parallel, we ranked the years from warmest to coldest, based on the temperature during the selected time window and divided the distribution in four quartiles. Lastly, we applied a χ^2^ test to evaluate the match between temperature quartiles and the anatomical extremes. This allowed us to assess whether extreme values in anatomical traits were associated with corresponding short-term extreme temperature fluctuations during the growing season.

## Results

### Tree-Ring Anatomical Traits

Dendroanatomical analysis allowed us to investigate xylem anatomical variations in Norway spruce and European larch over the last two centuries (**Figure 2**). CN was much higher in spruce than in larch rings while larch had larger cells (CD) in the first part of the ring (**Table 1**; **Figure 1**). Nonetheless, profile comparison evidenced some similarities between species. The largest CD usually occurred in sector 3 (i.e., at 1/4 – 1/3 of the ring profile) and slightly decreased until sector 7, however, toward the latewood, CD decline was sharper in larch. In the two species, CWT was similar in the first sectors, but larger in larch for the last sectors. CWT profile in both species increased around sectors 6–8. However, this increase was smoother in spruce than in larch, which evidenced a sharp wall thickening from sectors 6 to 9, while CWT in sector 10 was, on average, similar to sector 9 (**Table 1**). Descriptive statistics of anatomical series indicate that, in general, both CD and CWT MS and inter-tree correlation (Rbar) increased along the ring profile (**Table 1**).

**Table 1:** Descriptive statistics for mean cell number (CN), mean cell diameter (CD), and mean cell wall thickness (CWT) chronologies in spruce and larch over the period 1800–2011.

		Mean	*SD*	MS	Rbar
Spruce	CN	1936.37	439.76	0.126	0.188
	CD_s1	36.28	3.26	0.040	0.216
	CD_s2	38.09	3.40	0.040	0.223
	CD_s3	38.30	3.30	0.035	0.192
	CD_s4	37.46	3.06	0.037	0.197
	CD_s5	36.53	2.87	0.036	0.219
	CD_s6	35.33	2.82	0.039	0.222
	CD_s7	33.71	2.84	0.045	0.250
	CD_s8	30.79	2.79	0.056	0.297
	CD_s9	24.54	2.28	0.070	0.357
	CD_s10	17.04	1.01	0.042	0.324
	CWT_s1	2.81	0.11	0.025	0.023
	CWT_s2	2.85	0.11	0.022	0.012
	CWT_s3	2.88	0.12	0.023	0.012
	CWT_s4	2.93	0.13	0.025	0.026
	CWT_s5	3.01	0.15	0.028	0.053
	CWT_s6	3.12	0.17	0.031	0.061
	CWT_s7	3.28	0.20	0.041	0.102
	CWT_s8	3.56	0.28	0.064	0.207
	CWT_s9	4.11	0.43	0.094	0.369
	CWT_s10	4.51	0.46	0.093	0.410
Larch	CN	703.57	211.95	0.217	0.548
	CD_s1	46.90	5.15	0.093	0.184
	CD_s2	51.90	4.36	0.064	0.176
	CD_s3	52.27	4.72	0.080	0.283
	CD_s4	52.07	4.75	0.081	0.305
	CD_s5	50.92	4.75	0.080	0.290
	CD_s6	48.90	5.07	0.093	0.329
	CD_s7	44.59	5.35	0.115	0.269
	CD_s8	34.12	5.39	0.174	0.265
	CD_s9	19.19	2.40	0.127	0.223
	CD_s10	13.26	0.89	0.058	0.250
	CWT_s1	2.92	0.14	0.035	0.001
	CWT_s2	2.92	0.12	0.030	0.051
	CWT_s3	2.98	0.14	0.033	0.090
	CWT_s4	3.06	0.15	0.036	0.156
	CWT_s5	3.16	0.17	0.039	0.165
	CWT_s6	3.32	0.21	0.043	0.176
	CWT_s7	3.67	0.33	0.067	0.191
	CWT_s8	4.56	0.56	0.101	0.288
	CWT_s9	5.84	0.59	0.078	0.365
	CWT_s10	5.90	0.51	0.065	0.244

*CN statistics refer to the whole ring while CD and CWT are related to each of the 10 sectors. MS is chronology mean sensitivity; Rbar is the mean correlation between the series.*

Tree-ring anatomical profiles showed strong inter-annual variability, as exemplified in **Figure 1.** Within the same individual, tree-ring anatomy (profile configuration, cell number, cell size, and wall thickness within the sectors) can considerably change from years with cold (e.g., 1926, the first of 10 coldest June years in the 1926–2011 period) to warm (e.g., 1931, the first of 10 warmest June years in the 1926–2011 period) climate conditions.

### Ring Profiles and Monthly Climate Extremes

**FIGURE 3:**
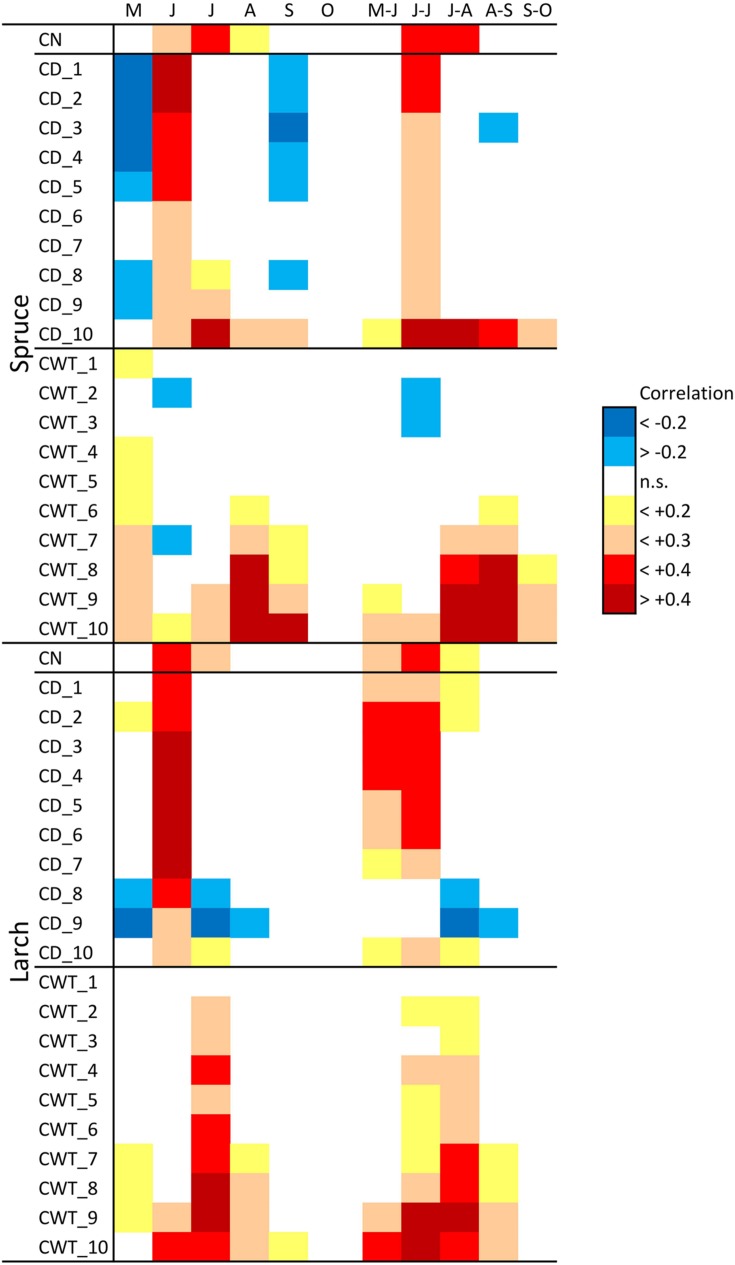
**Correlation between monthly (May–October) and bi-monthly (May–June to September–October) temperature during 1800–2011 and cell number (CN) chronologies, cell diameter (CD), and cell wall thickness (CWT) sector chronologies, in spruce and larch.** Correlation values are coded according to the chromatic scale on the right.

Considering climate variability with a monthly resolution during 1800–2011, CN in both larch and spruce was mainly related to June–July temperature (**Figure 3**). Furthermore, associations between climate and CD were quite similar in the two species. May temperature had a slight negative correlation with CD, while June temperature had a stronger positive correlation in all tree-ring sectors. Temperatures during spring and summer months were positively associated with CWT in both species, especially for the last sectors. In spruce, the highest correlations occurred for August and September temperature, while in larch they occurred for July temperature.

**FIGURE 4:**
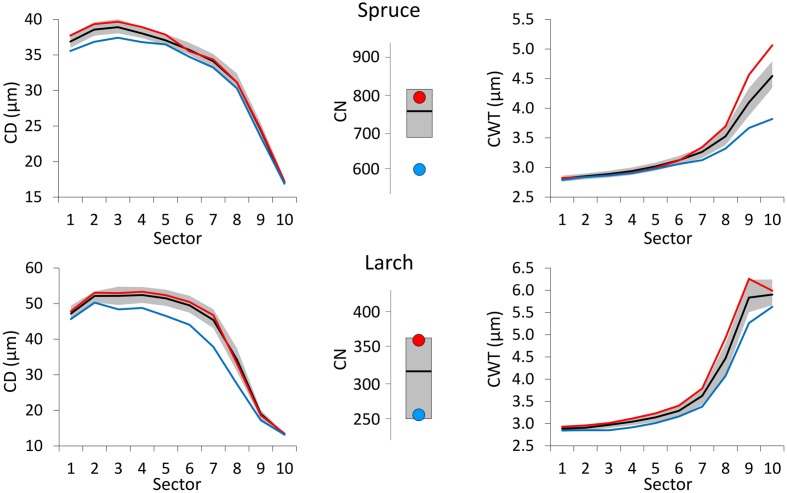
**Tree-ring anatomical profiles for mean CD (left) and mean CWT (CWT, right) along the 10 ring sectors in spruce (above) and larch (below), and plots of tree-ring cell number (CN, central).** Thick black lines represent the median profiles (median values, for CN) during 1800–2011, while gray areas define the 25th and 75th distribution percentiles. Red and blue lines (dots, for CN) represent the median profiles (values, for CN) during the 10 warmest and 10 coldest years respectively. Note different *Y*-axis scales.

Compared to the 1800–2011 median value, CN was larger in years with warm June–July, and smaller in cold years for both species (**Figure 4**). Deviation from the median was much stronger for spruce during cold years. Very cold June temperature induced a reduction in the CD of both species with respect to the median profile (1800–2011) and this deviation was more evident and consistent along the whole profile in larch. On the contrary, CD profiles during years with extremely warm June barely differed from the median ones. Cold conditions (during August and September for spruce, July for larch) affected CWT more than warm conditions inducing a clear divergence from the median in the last sectors toward the latewood, especially in spruce.

**FIGURE 5:**
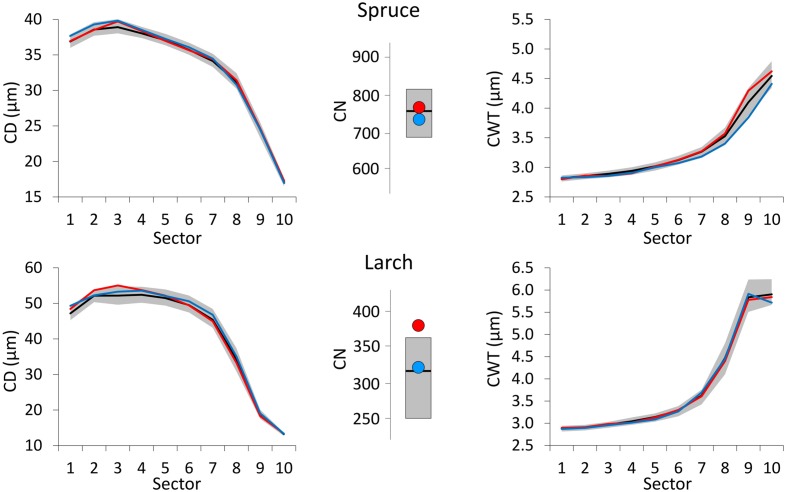
**The same representation as in Figure 4 but for the year after the extreme events**.

The year following the extreme event, all the anatomical profiles were similar to the median one (**Figure 5**). Only CN in larch, the year after an extremely warm June, was sensibly above the median.

### Intra-Ring Anatomy and Short-Term Climate Extremes

**FIGURE 6:**
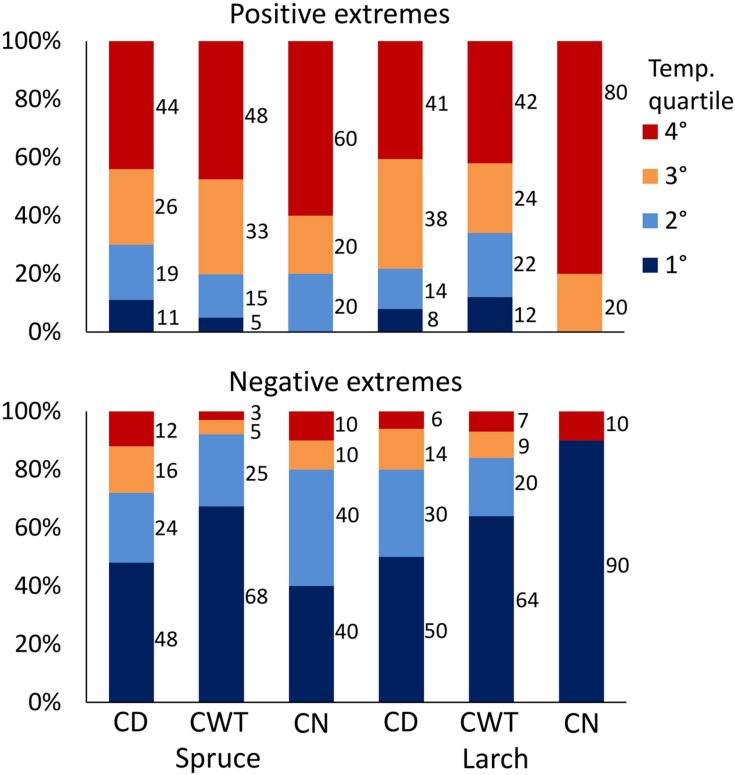
**Percentage of positive (above) and negative (below) extremes of CN, CD, and CWT in the two species falling within the four quartiles of temperature distribution for the period 1926–2011.** Temperature quartiles go from the coldest (blue) to the warmest (red). *p*-value of χ^2^ test was <0.001 for all 12 cases.

Investigation on climate association with ring anatomy at a finer scale (1926–2011 moving windows with 1-day step) showed a time shift of correlations along the ring profile, with maxima in the first sectors occurring earlier compared to maxima in the late sectors (correlations with daily maximum temperature are shown in Supplementary Figure S1). That is, detailed analysis was able to capture intra-ring differences in the xylem response to climate. After identification of the period with the greatest response to temperature in each sector, we scrutinized how anatomical traits were related to temperature conditions in that specific period. This analysis revealed that the 10 largest CN, CD, and CWT were usually associated with warm conditions, while smaller values were associated to cold (see Supplementary Figure S2 for results at the single sector level). On average, considering results for both species, all anatomical traits and sectors, 79% of positive extremes fell in years with temperature above the median, and 52% in the warmest quartile, while the lowest CD and CWT values mainly occurred during cold years (83% of negative extremes fell in years with temperature below the median, and 60% in the coldest quartile; **Figure 6**; *p* < 0.001 for all the cases, χ^2^ test). Looking at the single traits, CN extremes in larch were more strictly related to temperature than those in spruce. For CD and CWT, negative extremes were more related to cold conditions than the opposite. In both species, about half of the negative CD extremes occurred in the first (i.e., coldest) quartile of temperature distribution (**Figure 6**). About 2/3 of negative CWT extremes occurred in the first temperature quartile, and 92% (spruce) and 84% (larch) in years with temperature below the median.

## Discussion

The increasing occurrence and intensity of future extreme climate events is expected to have important aftereffects on forest ecosystems and the carbon cycle worldwide; this explains why a better knowledge on tree response strategies at different level is a scientific priority (Allen et al., 2010; Anderegg et al., 2015; Bussotti et al., 2015). This work represents a pilot study where we introduced some novel methodological and analytical aspects to gain a better understanding of the effects of climate extremes on xylem traits, especially in temperature-limited environments.

We sharpened the focus on several anatomical traits that are independent of one another and mostly related to the different xylem functions of water transport (CD) and mechanical support (CWT), with CN related to both. These traits are also related to different phases of xylogenesis: cell production via cambial division (CN), cell enlargement (CD), and wall thickening (CWT).

### Anatomical Response to Monthly Climate Extremes Over Two Centuries

Investigation on anatomical traits along tree-ring series allowed us to cover a long time period. In particular, we were able to assess the xylem-anatomical responses of two species to the 10 coldest and warmest years in the last two centuries, providing a robust assessment of the effect of extremes on xylem structure. Indeed, climate extremes are rare by definition, therefore studies not covering a long time span can hardly investigate more than one or very few events. This could hamper a complete mechanistic view of the cause and effect relationships, as the frequently idiosyncratic responses to rare events such as droughts or heatwaves has already been demonstrated by previous studies comparing two or more events (Lloret et al., 2011; Castagneri et al., 2015a; George et al., 2015).

Despite differences in their ecology and physiology, both species, typical of the subalpine environment, are well adapted to cold conditions (Tranquillini, 1979). Nonetheless, temperature and climate extremes influence wood formation at this elevation (Körner, 2006; Vaganov et al., 2006). With climate assessed at monthly resolution, in general we observed a rather similar response to mean and extreme events, with a common bias toward cold extremes, particularly evident for CD. This kind of unidirectional sensitivity to extreme cold seems in contradiction to the common knowledge of tree responses in temperature-limited conditions. However, this can be explained considering the above-mentioned main function of hydraulic transport related to the CD trait. Along and across the stem, monotonic change in lumen size has been reported to be almost universal within vascular plants, being strictly connected with stem elongation. It represents the most efficient anatomical adjustment to balance hydraulic path-length resistance with the progressive growth in height (Anfodillo et al., 2006; Carrer et al., 2015). The tight link between stem height and lumen size explains the relative unresponsiveness of CD to high temperature. Larger conduits would be unnecessary to efficiently transport water in trees with the same height but, on the contrary, they would increase the risk of winter embolisms (Mayr et al., 2006). Therefore, to observe a positive effect of high temperature on CD a fairly long series of warm years would be needed to permit the trees to grow taller and not just a single extreme event. On the contrary, low temperature in early summer can likely limit the mobilization and fixation of photosynthates (Petit et al., 2011; Körner, 2015), which affects the enlargement of primary wall and results in cells with reduced diameter.

Both the species and most of the anatomical traits did not show any carry-over effect respect previous-year extreme climate events. Previous growing season conditions can significantly affect tree growth of many conifer species, as demonstrated by several studies (Frank and Esper, 2005; Babst et al., 2013). Similarly, extreme climate events can leave a long-lasting legacy effect on the growth rates of many tree species, especially in harsh environmental conditions (Babst et al., 2012; Anderegg et al., 2015). However, most of past studies investigated just the tree-ring width, mostly related to cell number. Looking at different anatomical traits, it seems that larch and spruce trees close to the treeline did not present any carry-over effect in relation to unusual warm and cold short-term spells occurred in the previous year growing season. Probably the extreme events we defined were too short to induce inertial effects able to extend their impact beyond the short time span when they occurred.

Considering CWT, the impact of warm and cold monthly climate extremes seems overall quite balanced and more evident in spruce in the last ring sectors. This is likely due to the longer time of wall thickening phase in the xylogenetic process, especially in the last cells of the ring, and to the higher plasticity of this anatomical trait with respect to lumen size. The better capacity of tree-ring maximum density, a parameter highly related to CWT, to track temperature variability with respect to ring width (Briffa et al., 2002), which is closely correlated to CN, seems to be in line with these results.

### Anatomical Response to Short-Term Climate Extremes

In this study, we endeavored to assess the effect of short-term climate extremes on adult trees by investigating xylem anatomy at intra-ring level coupled with a high-quality daily-resolved climate dataset, an aspect still neglected in the current literature (George et al., 2015).

In general, CWT and CN extremes were more clearly associated to climate than CD, confirming what was observed with the lower-resolution monthly analysis. Difficulties in matching mainly CD extremes with the corresponding short-term climate variability might be related to year-to-year variations in cambial reactivation, and consequently in the timing of xylogenesis phases. Indeed, the peak of the 15-days window in climate sensitivity results from the simplistic assumption, common to many dendroanatomical studies (e.g., Liang et al., 2013; Castagneri et al., 2015b), that tree phenology is related to Julian days, i.e., anatomical responses to climate are fixed on the same days in all years. However, different phenological phases are usually tuned to the course of present or closely prior climate conditions (Seo et al., 2008; Rossi et al., 2014). The high-resolution time window that we introduced is likely a step forward for detecting short-term climate extremes effect on wood anatomy, but is still not enough to cope with the corresponding phenological plasticity in xylogenesis, and other approaches to detect the “key” time window during the season are advisable. CN, determined by cell division throughout the cambial activity period, and CWT, related to the wall thickening phase that can last up to 40–50 days (Rossi et al., 2008) seem to perform better in relation to the longer period of formation. In this case, inter-annual variations of xylogenesis phases were probably less appreciable considering that these traits integrate climate information on a longer time window.

The high significance of the results indicates that most of the extreme values in anatomical traits fall on the correct side of the temperature distribution (**Figure 6**). However, there is a margin of 0–34% of occurrences that did not match the expected temperature in their corresponding time window. Provided that short-term heatwaves or cold spells affect xylem structure only if they occur in (or immediately before) the period of active xylogenesis, in most of the events tree response could present a certain delay (Babst et al., 2012). Retrospectively identifying the cause/effect time lag together with a better focus on the past course of xylem phenology will surely improve the quality of the inferences. Moreover, the simultaneous effects of many climatic variables on growth could likely mask the impact of individual extreme climatic events on growth response (Ettl and Peterson, 1995). Lastly, detailed analyses on growth-climate interactions probably enhance differences in the individual responses related to microsite and surrounding conditions, genotypic variability, local disturbances, etc. (Carrer, 2011; Rozas, 2015), compared to extensive approaches that capture seasonal-level response of the tree population. These peculiar growth responses usually tend to converge, providing more consistent outcomes in the presence of a clear environmental factor limiting growth on a longer time scale.

## Conclusion

Despite the rising attention within the scientific community, and the prospected increasing role in future climate scenarios, short-term extreme events are still rather elusive. This is a challenge for the retrospective detection of such events in the tree-ring series and only the introduction of approaches that investigate different temporal scales could lead to progressive results. In this pilot investigation, we proposed some strategies for assessing the role of extreme events in a long-term perspective. Dendroanatomical measurements, with tree-ring sectors partitioning, and the use of high-quality daily-resolved *in situ* temperature records should go in this direction and has shown a good potential. Our analyses focused on a temperature-limited environment: here, the short-term extreme events detection appears rather challenging; dealing with other factors with a discrete nature (e.g., precipitation or winds) should be more straightforward.

Forest ecosystems are dominated by long-lived organisms, where extreme events can extend their effects from when they occur, even with a legacy of several years (Babst et al., 2012). There is much to be learned about the critical role of contingency and hysteresis in forest ecosystem response after climate extremes (Anderegg et al., 2015), and a better integration of empirical and modeling studies is needed, given that climate and vegetation models still do not realistically simulate most climate extremes and their effects (Reichstein et al., 2013). Wood represents an ideal archive to test for extreme events effects as it can provide a long temporal record; with tree-ring anatomy we have another powerful tool to improve our comprehension on such critical phenomena.

## Author Contributions

MC and DC were responsible for the study design, data analysis, and results interpretation. MC and DC provided the xylem anatomical data. MB analyzed and provided the climate records. All authors contributed to manuscript writing and revision and finally read and approved the submitted version.

## Conflict of Interest Statement

The authors declare that the research was conducted in the absence of any commercial or financial relationships that could be construed as a potential conflict of interest.
