# Supplementary material for: The Imprint of Extreme Climate Events in Century-Long Time Series of Wood Anatomical Traits in High-Elevation Conifers
Source: Front Plant Sci. 2016 May 18;7:683. doi: 10.3389/fpls.2016.00683 (PMC4870858; doi:10.3389/fpls.2016.00683)
Supplement: Supplementary file 1 [file Presentation_1.PDF]

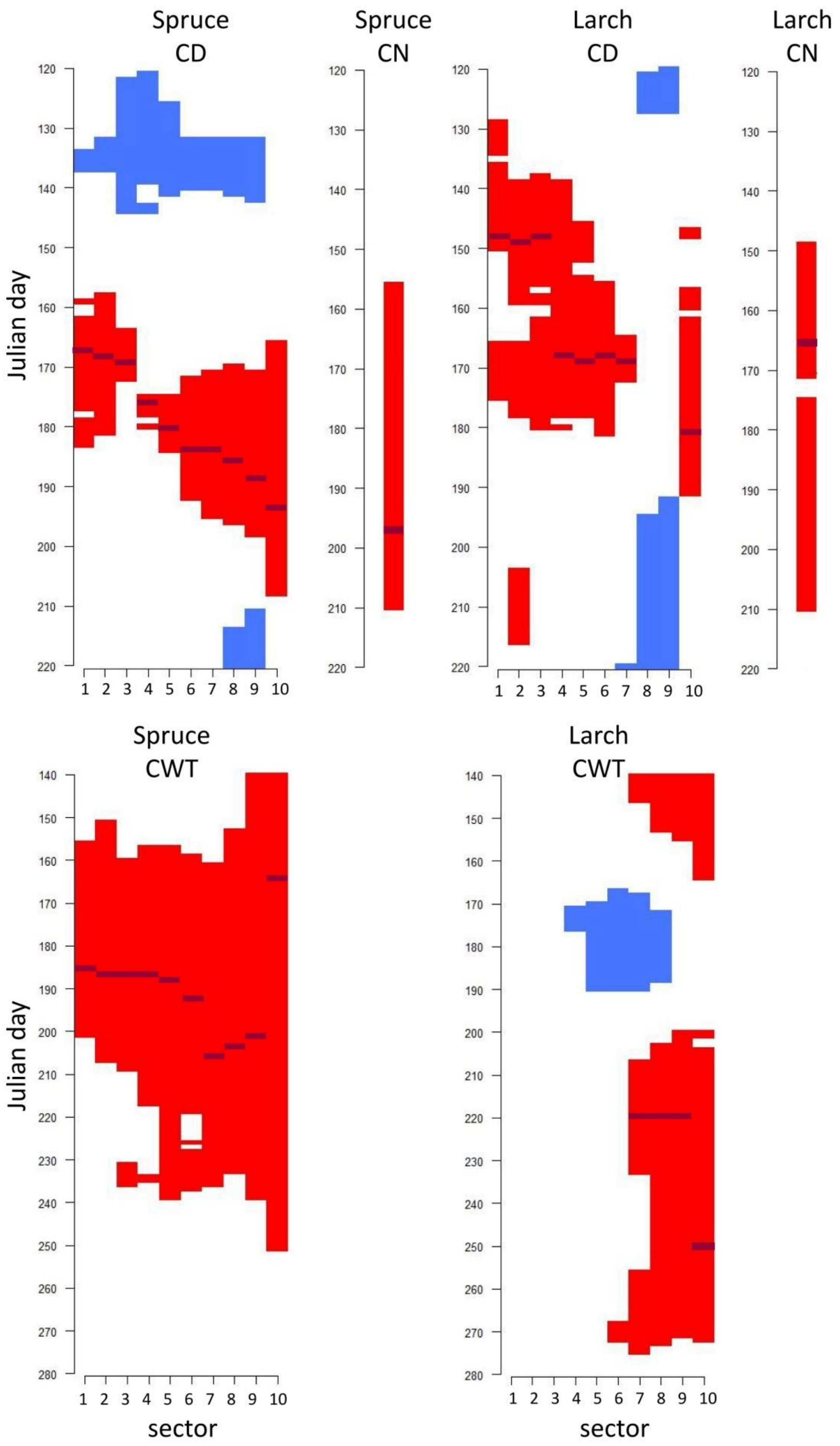

Figure S1. Correlations between mean cell number (CN), cell diameter (CD) and cell wall thickness (CWT) in each tree-ring sector (X-axis), with the maximum temperature expressed as a 15-day (CD) and 30-day (CN and CWT) running mean (1926-2011). Correlation coefficients are represented for each time window shifted at daily step. Red and blue coding indicates significant ($p \leq 0.05$) positive or negative correlation, respectively. Darker marks point out the time windows when correlation peaks.

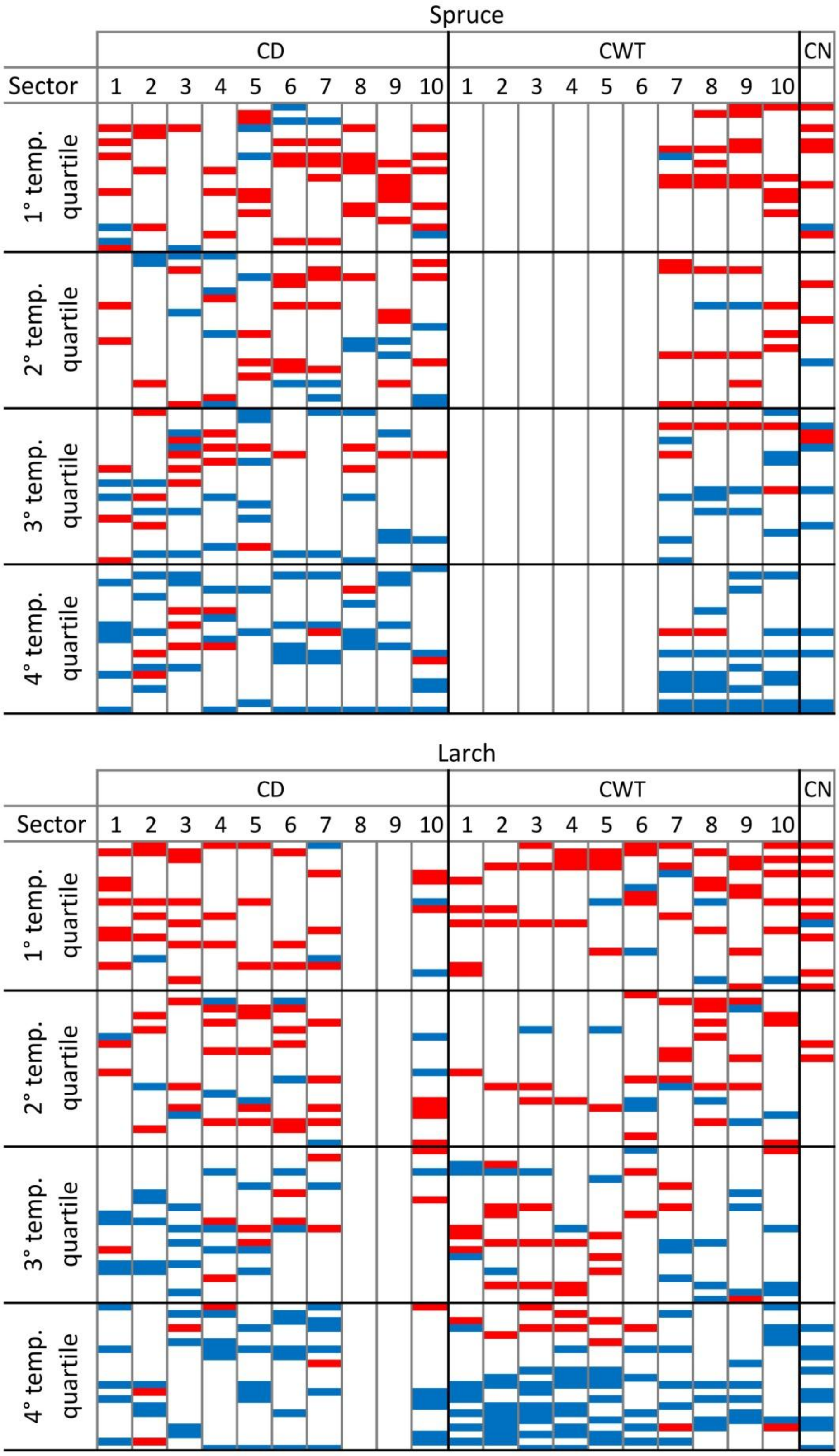

Figure S2. 10 high (red) and 10 low (blue) extreme values of cell diameter (CD), cell-wall thickness (CWT), and cell number (CN) in spruce (up) and larch (bottom), ordered according to the rank of temperature during the time window with the highest correlation with each parameter (as identify by moving window correlation analysis, see darker marks in figure S1), for the period 1926-2011. Empty columns correspond to the sectors that did not show significant correlation with temperature during spring-summer (see figure S1).
